# The Danger Model Approach to the Pathogenesis of the Rheumatic Diseases

**DOI:** 10.1155/2015/506089

**Published:** 2015-04-20

**Authors:** César Pacheco-Tena, Susana Aideé González-Chávez

**Affiliations:** Facultad de Medicina, Universidad Autónoma de Chihuahua, Circuito No. 1, Nuevo Campus Universitario, 31240 Chihuahua, CHIH, Mexico

## Abstract

The danger model was proposed by Polly Matzinger as complement to the traditional self-non-self- (SNS-) model to explain the immunoreactivity. The danger model proposes a central role of the tissular cells' discomfort as an element to prime the immune response processes in opposition to the traditional SNS-model where foreignness is a prerequisite. However recent insights in the proteomics of diverse tissular cells have revealed that under stressful conditions they have a significant potential to initiate, coordinate, and perpetuate autoimmune processes, in many cases, ruling over the adaptive immune response cells; this ruling potential can also be confirmed by observations in several genetically manipulated animal models. Here, we review the pathogenesis of rheumatic diseases such as systemic lupus erythematous, rheumatoid arthritis, spondyloarthritis including ankylosing spondylitis, psoriasis, and Crohn's disease and provide realistic approaches based on the logic of the danger model. We assume that tissular dysfunction is a prerequisite for chronic autoimmunity and propose two genetically conferred hypothetical roles for the tissular cells causing the disease: (A) the Impaired cell and (B) the paranoid cell. Both roles are not mutually exclusive. Some examples in human disease and in animal models are provided based on current evidence.

## 1. Outline of the Danger Model

**Figure 1:**
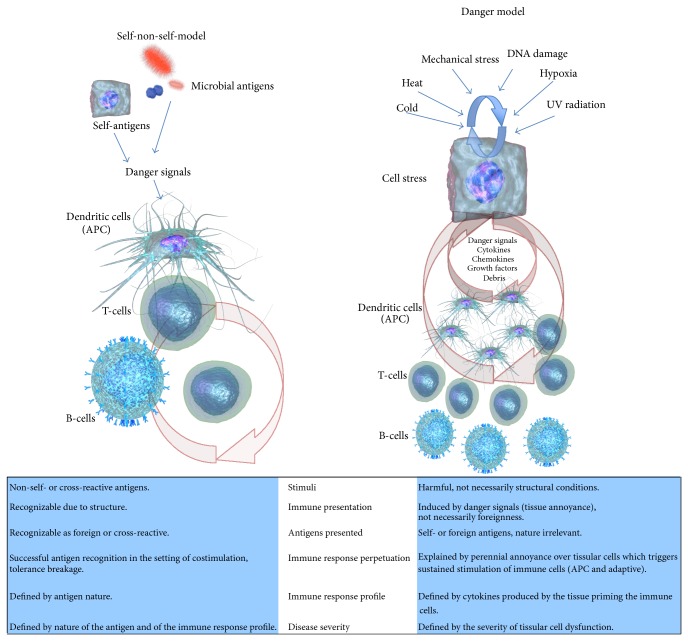
Basic comparison of the postulates between the self-non-self- (SNS-) model and the danger model (DM). In the SNS-mode, the triggering stimulus is the antigen which is by definition foreign, or, if endogenous, it is mistaken as foreign; once the antigen specific cells have been primed, the persistence of the immune response depends on the perpetual presence of an antigen and for the case of an autoantigen on its expression where it can be detected and processed by antigen presenting cells to T-cells; the severity of the immune reactions depends on the nature and amount of the antigen and the type of immune response it settles on. In the case of the DM the initial step is a scenario of disturbance within the tissues which can be explained by both biological or physical aggressions, the disturbed tissular cell signals to the local antigen presenting cells, and, as the aggression becomes more chronic the tissular cell communicates directly to T- or B-cells; the perpetuating cycle for the case of chronic autoimmune diseases relies on the repeated disturbance of the tissular cells by the annoying stimuli and self-proteins are recognized eventually as antigens due to the enhanced antigenic presentation costimulation upregulated by the soluble factors released by the stressed tissular cells. The severity of the immune reaction depends on the intensity and frequency of the disturbance that the stimuli infringe in the tissular cells.

The danger model (DM) was proposed by Poly Matzinger as an alternative (or complement) to the traditional self-non-self- (SNS-) model [1]. The DM postulates that the immune system decides to start an immune response if a potential threat is able to induce harm in the tissues, in counterpart to the SNS-model where foreignness is a fundamental precondition. Matzinger has explained the rationale of her model in several papers [1–3], including a historical perspective [4, 5] linking the DM to the SNS; we will only give a brief overview to set the context of our paper (Figure 1).

In the initial conception of the SNS, Burnet proposed that the B-cells carried multiple antigenic receptors specific for one epitope. The binding of these receptors to its specific ligand triggered an immune response, and it was assumed that this binding sent a signal to the B-cell (signal 1). Later, Bretscher and Cohn incorporated the T-cell in their associative recognition model [6]; on it, the activation of B-cells required not only the signal 1 but also the help signal from another cell (helper T-cell) also specific for the same antigen which provided an additional signal (signal 2); otherwise, the antigen-primed B-cell, if not rescued from the T helper cell, would die. Eventually it was found that also the helper T-cells require a second signal in addition to that provided by the antigenic recognition; this signal was named co-stimulation, and it came from antigen presenting cells (APC). APC are able to process and present antigens from phagocytized material, but lack antigenic recognition and therefore specificity. The decision of an APC to either upregulate or not the co-stimulatory molecules at the time the antigen is presented, defines the fate of the primed specific T-cell (stimulation, anergy, apoptosis, differentiation); yet the cell that decides it (the APC), is unaware of the self-non-self-status of the presented antigen. This central role for an antigen-undiscriminating cell in the outcome of an immune response posted a major challenge to the logic of SNS-model.

The discovery of pattern recognition receptors (PRR) by Medzhitov et al. [7], gave the APC a certain SNS-discriminating personality, because the PRR were thought to target specifically highly conserved structures from microorganisms. In that perspective, PRR could warrant foreignness (non-self-) discernment as a criterion for immunogenicity; self instead was spared. However, shortly after the PRR and their specificities were characterized, it was shown that PRR also bind, recognize and are activated by endogenous (self-) structural components in a physiological basis [8–15]. PRR cannot distinguish self from nonself either, nothing does.

The DM however does not require SNS-discernment; it solves this apparent lack of control of the immunogenicity because it transfers the control of the immune response not to the mere antigenic recognition but to the prerequisite of harmful conditions inducing the activation of the cells lying within the tissues. In that command, the tissular cells require the presence of harmful conditions as a critical step to stimulate immune cells to start an immune response. In this perspective, the tissular cells become proactive elements enabled to communicate with the local immune cells (i.e., dendritic cells) and establish their status of wellbeing/suffering. The DM postulates that the presence of disturbance (stress) or damage within the tissue structure triggers a series of mediators released from the tissular cells that activates the APC (or others) to up-regulate costimulatory molecules and eventually prime specific T-cells. Quiescent tissues, on the other hand, are tolerogenic.

The DM gives a holistic approach to the immune system as a simple integrant in tissue homeostasis extending it beyond the antigenic recognition. An updated review of the mechanisms involved in the tissue-over-immune-response control have been detailed by Matzinger and Kamala recently [16]. Conceding a commanding role for the tissular cells, the autoimmune scene gains several potential actors which may play undisclosed roles that will fill some of the inconsistences that currently trouble us.

The SNS-model requires an antigen, while the DM requires an abnormal stress signal. In the setting of an immune response if there is a danger signal, any protein processed and recognized is an antigen because the avidity of the T-cells is increased by a cocktail of stimulating cytokines, they are unable to discriminate whether the recognized protein explains the damage, they assume the connection, if a protein is abundant in the setting a tissue harm, probably there is a connection.

When microorganisms cause harm, a non-self-component exists in the scenario. The immune response preferably will target non-self- over self-antigens and the incipient autoimmunity will be eventually controlled. On the other hand, when harm is explained by aseptic cellular dysfunction (cold, mechanical stress, and hypoxia) the tissues will deliver the danger signal as a result of cell suffering, the presented antigens will be by definition self, and tolerance will eventually be broken because this type of cellular distress repeats itself in an incompetent cell. From this point on, a connection is created; there will be a link between the insulting stimuli (cold, mechanical stress, and hypoxia) to the inflammatory response and the immune aberration. The severity of the abnormality will be explained by both: the intensity of the stimuli (i.e., UV radiation in Lupus, cold in chilblains) and the degree of cellular dysfunction conferred by its impairing genome.

In multicellular complex organisms, the terminally differentiated cells adopt a wide diversity of phenotypes. As a particular stem cell matures and differentiates in both its structure and its function, it prepares itself to the expected harms it will face (infection, mechanical demand, and cold). The cells are programmed to endure to their environment, to choose the adequate response to overcome the threats, and to prepare their own healing; however under certain conditions defective genes have been described that avoid the proper functioning of the cell. We could think of dissimilar cells like the keratinocyte and the osteoblast, how different their local environments are, and also the threats they face. We could think of dissimilar cells like the keratinocyte and the osteoblast, how different their local environments are, and also the threats they face, how specialized these cells are, the number of specific stimuli they have to respond to, and the number of particular proteins and compounds they are able to produce in consequence. Could not be in the moiety of such specialization, in the mastering of its environment, the ability to tailor its local immune responses? Or, on the other hand, from the antigen-driven SNS-perspective if the T-cell is in command, how can a T-cell be instructed to both recognize an antigen and also to suit a specific immune response to every scenario, how can the thymus anticipate in which tissue that antigen-T-cell receptor (TCR) encounter will take place, and how can that instruction be? Likely the selection of the immune response effector mechanisms, healing, and tolerizing processes result as consequence of a dialog between local cells and T-cells, chances are that the T-cells are listening, and they are not in command.

## 2. The Danger Model Boundaries

DM is frequently associated to PRR and innate immune response; also it is commonly linked to the recognition of microbial structures (danger signals) by immune cells through antigen nondiscriminating receptors [12, 17, 18]. Mostly, the DM is connoted as an array of archaic or rudimentary mechanisms. For most conceptual frameworks DM is subordinated to the regulation of the adaptive immune response, which we consider to be more complex, modern, and versatile.

The DM is mostly limited to the recognition of harmful situations. It is restricted to be an enhancer triggering inflammation or a costimulation inducer in the antigenic presentation, but not to command an already established immune response. This perspective remains antigen-centered; once the danger recognition causes an antigenic recognition and costimulation catches on, the tissular cells open the door for the professionals and afterwards become bystanders or victims of the resultant inflammation. This casing of the DM as a mere detector is probably a hyperbola to make it fit under the perspective of SNS and make it politically correct. At this point the idea that an autoimmune disease can be explained solely by a tissular cell abnormality without a relevant role for the immune system seems to say the least unlikely; however, as will be mentioned later in the text, current evidence shows the opposite.

The DM is neither limited to the innate immune response mediators nor its cells; it is not necessarily primitive or subordinated. Tissular cells (keratinocytes, adipocytes, chondrocytes, etc.) may interact directly to T-cells or B-cells bypassing APC because they produce relevant fancy high-profile immune mediators. Tissular cells are in the position to control processes so critical as the differentiation of T- or B-cells to specific subtypes or downregulate them in many different ways [16].

Keratinocytes, for example, produce type-I interferons [19, 20], IL-1 [21], IL-6 [22], IL-8 [23, 24], IL-17C [25, 26], IL-18 [27, 28], IL-20 [21], IL-24 [29], IL-25 [21], and IL-33 [30] and also several chemokines [31–33] and growth factors, so why do we need to limit the danger signals to innate immune response? Several other tissular cells such as fibroblasts, chondrocytes, and epithelial cells bear similar arsenals. Therefore these cells can also communicate directly to cells of the adaptive immune response; they prime them, stimulate or inhibit them, and control their differentiation. In the DM the key players are indeed the tissular cell, the mediators produced by it, and the effect of these mediators in the environment, regardless of the type of immune response involved.

## 3. Evolutionary Vision of Danger Model

The concept that the cell should perceive the danger in its environment is understandable in the context of its eternal quest for survival, and the fact that the adaptive immune response is a more recent evolutionary advance does not implicate that its presence precludes the tissular cells from being the commanders. In evolution a basic premise is the structure-function combination; structures remain only if they are functional. Evidently tissular cells have to exist to maintain structural features of the organism, but they did not had to preserve, almost intact, the intricate signaling systems that communicate harm or wellbeing. These signaling cascades are conserved all over from the stimuli, the receptor, and the signaling cascade down to the effector mechanism; the diversity of the natural potential dangers explains the existence of specific signaling cascades triggered by a diversity of harmful conditions (i.e., heat, osmotic changes, ultraviolet radiation, mechanical strain, etc.).

The elicited responses from bacteria and human cells to several of these threats are very similar; in fact, the involved proteins in these responses are preserved between prokaryotes and humans and exhibit a high phylogenetic preservation. These signaling pathways include responses to heat [34], cold including the cold shock proteins which evolved into cold shock domain in eukaryotes [35], DNA damage and repairing mechanisms [36], apoptosis [37], aging and inflammation throughout nuclear factor kappa-light-chain-enhancer of activated B-cells (NF-*κ*B) [38], protective mechanism against oxidative stress [39], hyperosmolarity [40], autophagy and transcription [41], and prime intracellular signaling pathways such as G-protein [42] and tyrosine kinases [43]. Therefore ancient protective mechanisms remain basically unchanged and represent relevant players in mammal and human defensive and housekeeping cellular processes including intercellular communication. If the adaptive response had provided a solution for the diversity of potential harms, what is the logic of preserving these tissular cell sensors? Very likely the adaptive response remains subordinated to these ancestral mechanisms, and we could think of the adaptive immune response as the microphone, not as the voice.

To exemplify how evolution defines development and control we could exemplify the central nervous system. What is in your opinion more complex, the brain cortex or the brain stem? Undoubtedly it is the brain cortex, which has provided the evolutionary advantage of the intelligence to the mammals and particularly the humans. However, we can survive without the brain cortex, but not at all without the brainstem; the brain cortex is a mere scalar extension of the brain stem.

Immune system evolution and diversification rely on basic ancient mechanisms which have developed altogether trying to resolve harm and threats to tissular cells (for most cases the ones endangered); these mechanisms incorporate new elements, cells, and mediators in a progressive fashion, but its fundamental structure has probably changed a little from its basics. Our fascination with the concept of the antigenic recognition as the onset of any immune response gave to the antigen specific cells a primal spot to drive our understanding assuming that the threats are external and neglecting the role of immune system in tissue homeostasis under aseptic conditions.

In evolution, similar functions are accomplished throughout different strategies but in increasingly complex scalar models. Competent nervous systems were there before the brain developed the cortex (think of the complex behavior of bees) and competent immune systems were there far before T- or B-cells appeared in the jawed fishes (think of the septic environment of several invertebrates, insects included). Although autoimmunity cannot be presumed in an organism lacking self-discernment, organic damage associated to an exaggerated inflammatory response due to failure of normal immune regulators is indeed detected in invertebrates such as the fruit fly,* Drosophila*.

These alterations in* Drosophila* include scenarios not unfamiliar with human disease such as abnormal interaction with commensal flora causing uncontrolled intestinal inflammation [44–46], chronic inflammation associated to carcinogenesis [47, 48], defective immunoregulation in the TGF-*β* cascades affecting wound healing [49], excessive uncontrolled inflammatory responses [50], structural mutations in structural proteins like lamin which cause encapsulation by hemocytes, therefore presenting the link between mutations inducing cell dysfunction and its translation into proinflammatory environments [51]. We can also see inadequate responses to stress oxidative responses which associate with mutations resulting in chronic inflammation; also, in the case of mutation of the Parkin gene it induces mitochondrial dysfunction and upregulation of genes of the innate immune response, degenerating the flying muscles [52, 53]. Therefore autoimmune-like phenomena precede the existence and function of the adaptive immune response; and, likely some mechanisms explaining autoimmunity in human beings are linked to tissular cells and disorganization of danger signaling/perceiving systems and not only to innate immune response. Abnormalities in ancient survival mechanisms could therefore explain chronic autoimmunity in humans as well; adaptive immune cells obviously could add more instruments to the orchestra but only to play the same song.

## 4. Danger Model and Rheumatic Diseases

As we look into the histopathological picture of the inflamed tissues in the immune mediated rheumatic diseases, it is logical to assume that immune cells are responsible for the aberration in the inflamed tissues, and it is logical, too, to assume that the dysfunctional behavior of these immune cells is the key pathogenic process, but what is really the role of the inflamed tissues? If we (our society) were seen under a microscope, the observer could deduct several concepts when looking at our ordinary conflicts; likely he or she would assume that fireman causes the home to fire since most of the time when a fire is detected eventually the presence of the firemen would be advisable. What can we conclude when we see the densely packed lymphocytic infiltrates lining under the dermis of psoriatic or lupic patients? What happened before they got there? Do we know it? Are these infiltrating lymphocytes responsible for the abnormality, or are they simply taking the call? Where the problem does really lies? In the abnormal call from an abnormal tissue to a normal lymphocyte or in the normal call to an abnormal lymphocyte? Both? Furthermore these infiltrates are replicated in animal models with the mutation of genes affecting tissular cells' wellbeing but with no clear role in immune regulation (vide infra).

The antigenic responses observed in autoimmune rheumatic diseases frequently target harmless housekeeping proteins. For a stressed cell, its physiologic response to the harm involves the upregulation of several of these housekeeping, stress-induced proteins. These proteins are therefore abundant in stressed cells and in their vicinity. When antigen processing cells are recruited due to the tissular stress response, very likely the upregulated proteins will be ingested and presented to immune competent cells altogether with the adequate costimulation which is induced by the danger signals from the tissular cells. Immune targeting to housekeeping proteins in chronic autoimmune diseases is no better exemplified than that to heat-shock proteins (hsp).

Traditionally immune response toward hsp in rheumatic diseases was assumed to be the consequence of hsp phylogenetic preservation and putative cross-reactivity toward bacterial hsp. The reactivity toward hsp is assumed as a sequel to either a previous infection or the habitual commensal contact [54–57]. In RA, antibodies against hsp40, hsp47, hsp60, hsp70, and hsp90 have been described [58] and humoral and cellular immune reactivity to several hsp have been reported as well in SpA patients [59, 60]. The immunoreactivity to hsp is not limited to rheumatic diseases and is present also in unrelated diseases such as diabetes mellitus [61] or schizophrenia [62]. Interestingly, although the immune response in several models of arthritis in rodents reacts with hsp, direct immunization with hsp has repeatedly failed to induce arthritis [63]. In recent times more than its putative cross-reactivity, is the biology of human hsp the one that has become a matter of interest. Citrullinated human hsp90 is linked to interstitial lung disease in patients with RA [64] and, recently, it was shown that the humoral immune response in patients with SpA targets human hsp-60 and not the bacterial one, therefore challenging the cross-reactivity scenario [27] and suggesting a direct role for that protein in the inflammatory process. The increased presence of hsp in the RA synovium has been interpreted as a potential door for them to become autoantigens, that is, hsp90 as a ligand to TLR2 [65] or hsp22 binding to TLR4 [66], but what is the real situation inducing the upregulated expression of these hsp in the synovium in the first place? Is it assumed that they are upregulated to become autoantigens? Most likely they are upregulated because synovial cells (i.e., fibroblasts) are stressed and hsp are fundamental chaperones if the cell damages or stresses; and the inflammatory response is associated to this stress and not to the presence of the hsp. Hsp are antigenic because they are abundant in the context of this cell stress; then they are trimmed, processed, and presented, and sooner or later they become recognized altogether with the cocktail of danger signals enhancing antigenic presentation and costimulation. As shown in animal models, they are not on their own antigenic (as DNA).

## 5. Theoretical Approach for Tissular Cell Dysfunction as an Etiology in Rheumatic Diseases from the Perspective of the Danger Model

In this paper, we propose that chronic tissular cellular dysfunction is the prerequisite for autoimmunity to be settled. We propose that this nonlethal tissular dysfunction drives the cells to a status of “perennial annoyance.” This perennial annoyance is caused by continuous disturbing stimulation, which is physiologically expected in this tissue. This annoyance will eventually result in a sustained proinflammatory response from the tissue itself, and it is within the course of this chronic and cyclic response signaling process, where the autoimmune inflammatory process is subsequently induced and defined. This perennially annoyed status of the tissular cells mediates indeed the activation of the innate immune cells, also explains the induction of costimulation in APC cells, and eventually stimulates the antigenic presentation of self-upregulated tissue homeostatic mediators (counteracting the stimuli). These annoyance-induced self-proteins are eventually recognized as antigens by T-cells, and—probably the most emphatic asseveration in this paper—we propose that the tissular perennial annoyance indeed mandates the fate and phenotype of every chronic immune response even in the stages after the T-cells and the rest of adaptive immune response cells have been primed and differentiated and that the persistence of the inflammatory process has little to do with the subsequent antigenic recognition and binding, but does indeed rely on the dangerous cocktail of mediators that after a new challenging stimulus (cold, ultraviolet radiation, mechanical stress, etc.) reinforces the perennial annoyance in the tissular cells.

Therefore we consider that the DM can explain a significant proportion of the pathogenesis of autoimmune (or autoinflammatory) rheumatic diseases from the perspective of this tissular perennial annoyance. We propose two models strictly attached to de DM logic in order to explain it: the first model is the impaired cell and the second is the paranoid cell.

### 5.1. Model A: The Impaired Cell

For this model, the basic feature is a structural or metabolic impairment in the tissular cell. This impairment limits the capacity of the cell to undertake its physiologic functions or to respond to the normal stressing factors expected by its place in the body. The cell adapts as far as its impairment allows it, but eventually the dysfunction becomes evident, the cell is stressed, and it enters into the perennial annoyance status. We propose several potential scenarios exemplifying tissular cells with impairments to deal with specific aggressions that eventually generate inflammatory environments that lead to chronic immune responses.

#### 5.1.1. The Sun-Burned Defective DNA Repairer Keratinocyte in SLE

Inflammation of the skin is frequently seen in patients with SLE; specifically, acute cutaneous lupus relates to sun exposure, primarily UV radiation. UV radiation is a major threat for DNA; it is therefore expected that the naturally sunlight-exposed keratinocyte should be fitted to overcome that everyday induced DNA damage. DNA repairing mechanisms are numerous, involving a series of sequential enzymatic chains—which are critical in their roles—and, unfortunately, polymorphisms may generate hypofunctional or defective enzymes. In this scenario of defective DNA repairing, DNA damage could not reversed, and DNA integrity is critical to warrant genome functionality and cell homeostasis. DNA housekeeping represents an incessant time- and energy-consuming task for the cells.

Defects in DNA repairing mechanisms have been reported in SLE patients [67–75]. This defective DNA repairing has been implicated with the generation and accumulation of nuclear material, which is potentially antigenic. But would not the impairment to repair the damaged DNA induce a perennial stressed status in the keratinocyte? What drives the proinflammatory engine, the abnormal genetic material itself—and its potential antigenic nature—or the perennial annoyance of the cell? What is the real critical step for the antigen to be recognized and prime an immune response? Is the immune response explained by an abnormal (antigenic) recognition or is simply the keratinocyte upscaling its unconformity?

Defective DNA repairing as a relevant pathogenic mechanism explaining the induction of SLE can be confirmed in the Dnase1-deficient mice [76]. These mice replicate cardinal clinical and serologic features of SLE, and, in this controlled scenario, the complete picture is explained by the deficiency in one gene, which is a DNA housekeeping molecule, not an immune related one. Several aspects in this model are worth considering: first, the fact that both, the homozygous (Dnase1^−/−^) and the heterozygous (Dnase1^−/+^) deficiency induce the disease although the frequency and tittering of antinuclear antibodies are higher in the homozygous KO opens the possibility that partial defects and not necessarily deletions could induce the SLE phenotype; second, several specific SLE antinuclear antibodies were detected, such as double-stranded anti-DNA (only observed in the Dnase1^−/−^) and anti-Sm suggesting that the mere defective maintenance of DNA (no immune deregulation, no cross-reactivity) is being able to induce them; and, third, the mice developed glomerulonephritis therefore linking the mere defective DNA conservation to several key pathogenic manifestations in the human disease. In this same study the authors demonstrate a significant reduction in the activity of Dnase1 in human patients with SLE if compared to healthy controls. We can infer that the involvement of the adaptive immune response cells is simply a cascade of events precipitated by the DNA damage response.

An additional link between defective DNA handling and SLE can be seen in the Aicardi-Goutières syndrome (AGS). AGS is characterized by familial encephalopathy, calcification of basal ganglia, and cerebrospinal lymphocytosis; additionally, it shares some features with SLE [77] such as the involvement of interferon-alpha [78] and it also causes chilblains [79]. The AGS is caused by the mutation on any of the 3 domains of the H2 ribonuclease [80], in DNA exonuclease 1 (TREX1) [81], the sterile alpha motif domain and HD containing protein 1 (SAMHD1) [82], or adenosine deaminases acting on RNA (ADAR1) [83]. TREX1 has been already implicated as a susceptibility gene in SLE [84]. Eventually it became evident that patients with AGS—irrespective of the mutant causing enzyme—demonstrated SLE clinical and laboratory features such as positive antinuclear antibodies (including anti-DNA), leukocytopenia, thrombocytopenia, arthritis, and oral ulcers [85, 86].

In our opinion, as it happens with the Dnase1 model, it is of primary relevance how a single genetic mutation is able to confer a SLE phenotype. An hypothetical approach for the link between autoimmunity and the deficiency of these enzymes in AGS is related to an increment in the interferon type-I response [87, 88]; nevertheless the intrinsic physiological role of the AGS enzymes is the housekeeping care of nucleic acids [89–91] and they have no physiological relationship to the interferon production; defective function of the AGS conferring enzymes increases interferon production but not by a direct stimulus; TREX deficiency results in the ATM-dependent DNA damage checkpoint [92, 93] because single-stranded DNA accumulates in the cytoplasm where TREX normally resides.

**Figure 2:**
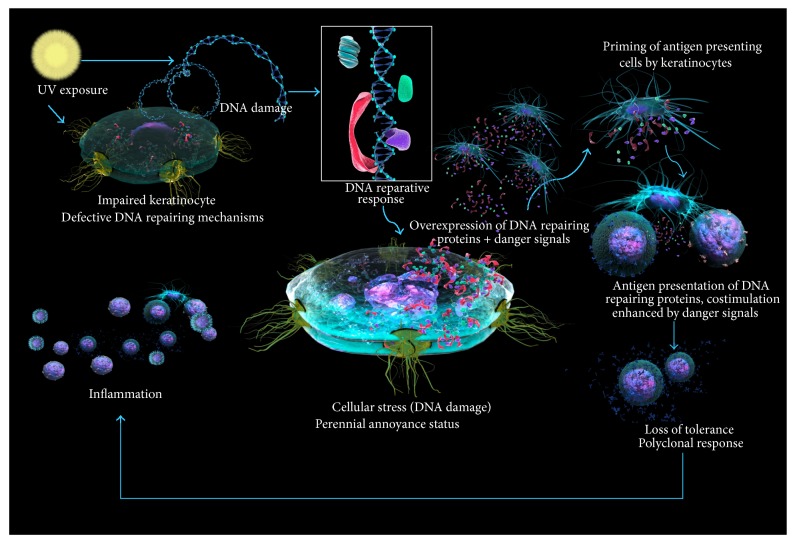
The sun-burned defective DNA repairer keratinocyte in SLE. The exposure of the keratinocyte DNA to UV radiation infringes DNA damage, which cannot be normally repaired because of faulty enzymes. DNA repairing proteins are upregulated and therefore presented as antigens; in the stressed context costimulatory molecules are upregulated and an autoimmune response toward nucleoproteins is settled. Repetitive cycles of UV radiation perpetuate the immune process because the tissue is harmed again and releases danger mediators.

DNA damage is an everyday fact and it induces inflammation [94–96] but also a DNA reparative response [97–100]. In physiological conditions, damaged DNA is repaired and the consequent inflammation fades; however, in a defective DNA repairing scenario (i.e., impaired keratinocyte), a perpetual inflammatory status could be eventually settled. Repeated efforts from the keratinocyte to maintain its DNA integrity and physiological function eventually fail. A defective repairing pathway could be compensated by others, and the repairing proteins will be upregulated beyond physiological levels that altogether with the cell stress scenario will make great antigenic candidates out of them. Once the tolerogenic nature of the tissue is lost and the danger signals spread up, an unspecific mononuclear infiltrate lies around immune activated keratinocytes, and the role of the immune competent cells is far from being understood; likely those cells are just answering the call; the caller has the structure to congregate them (Figure 2).

Keratinocytes under UV irradiation secrete a diversity of proteins [101, 102]: some of them are linked to the reparation of the DNA and others carry proinflammatory actions rendering the keratinocyte capable of activating local dendritic cells and also adaptive immune response cells. Since the reason for the call is sustained stress, a quest for an antigen to be detected is a must for antigen specific cells. However in this milieu of stress induced immune cell activation, with the cocktail of costimulatory molecules upregulated by danger signals in the APC, eventually a self-antigen becomes recognized. DNA metabolic pathways are targeted, maybe because some defects among those pathways are detected as failures.

The DNA damage response upregulates several DNA repairing enzymes. It is therefore probable that, in the cytoplasm of these cells, DNA and RNA repairing or keeping enzymes or chaperones are abundant, altogether with histones and other DNA packing and unpacking proteins, and also the synthetic machineries of nucleic acids and nucleic acid associated proteins, such as U-RNP and its subunits (including Sm), and proteic synthetic enzymes are required. At the same time the cell is repairing itself; it is also calling for help as can be inferred by the transcriptomes of UV-damaged keratinocytes. It would be interesting to find out whether relevant antigen targets in connective tissue diseases (topoisomerase, U1-RNP, Ro, La, etc.) actually play a role in the process of DNA reparation.

#### 5.1.2. The Hypoxic Fibroblast in Rheumatoid Arthritis (RA)

Pathogenesis of RA is complex and likely includes several physiologic abnormalities aside from immunological abnormalities. Hypoxia has been recalled as a potential mechanism for RA since a long time ago [103]; abnormally low levels of PO_2_ in the periarticular tissues and in the synovial membrane were described by Doust and eventually confirmed by Ng et al. [104]. The severity of the synovial hypoxia in patients with RA correlates with the levels of inflammatory cytokines and also with the density of the immune cells in the membrane implicating a role for the hypoxia in the pathogenesis of the disease. Rothschild and Masi in 1982 also correlated the hypoxia with the vascular proliferation [105], another cardinal feature at early stages of synovitis. Although the genesis of hypoxia in the synovial membrane is unclear, vasoconstriction due to upregulation of angiotensin and angiotensin converting enzyme is a candidate mechanism [106]. Interestingly hypoxia is also a feature of animal models of RA such as collagen induced arthritis, and Jeon et al. [107] demonstrated that in this model hypoxia precedes inflammation; the hypoxia was inferred by the expression of hydroxyprobe-1 which was detectable 1 week earlier to the inflammation.

Hypoxia was eventually linked to several critical pathological processes of the synovitis [108, 109]. Hypoxia induces a wide array of inflammatory genes in macrophages [110]. Allen et al. linked the hypoxia in the synovium to the induction of superoxide radical generation [111] and Stevens et al. linked hypoxia to both inflammation and neovascularization [112]. Additionally a secondary hypoxic-reperfusion cycle [113, 114] involving the Von Willebrand factor and reactive oxygen radicals release [110, 115, 116] was reported; this cycle has been also related to the expression of NF*κ*B and upregulation of ICAM-1, very likely enhancing several inflammatory mechanisms [117].

Hypoxia also induces several cytokines in the rheumatoid synovium, which presumably play a role in the induction and perpetuation of the inflammation. Some of the induced cytokines are the stromal cell-derived factor 1 (CXCL12), the vascular endothelial growth factor (VEGF) [118–121], TGF B, IL-1, and TNF-*α* [122], IL-20 [123], and IL-8 [124, 125]. Also hypoxia induces the expression of COX 2 [126] in fibroblast-like synoviocytes and upregulates MMP-1 and MMP-3; meanwhile it inhibits TiMP-1 [127]. Some redundancy in the pathways is assumed since TNF-*α* and IL-1 themselves modulate the production of VEGF in vitro.

Hypoxia also induces the expression of hypoxia-induced factor alpha (HIF) [128]. HIF is upregulated in synovial macrophages of RA synovium in comparison to that of osteoarthritis (OA) [129], and its expression induces the production of VEGF and platelet endothelial derived cell growth factor (PD-ECGF) [130] relating it to the vascular proliferation observed in the synovium; HIF is also connected to the production of MMP-3.

**Figure 3:**
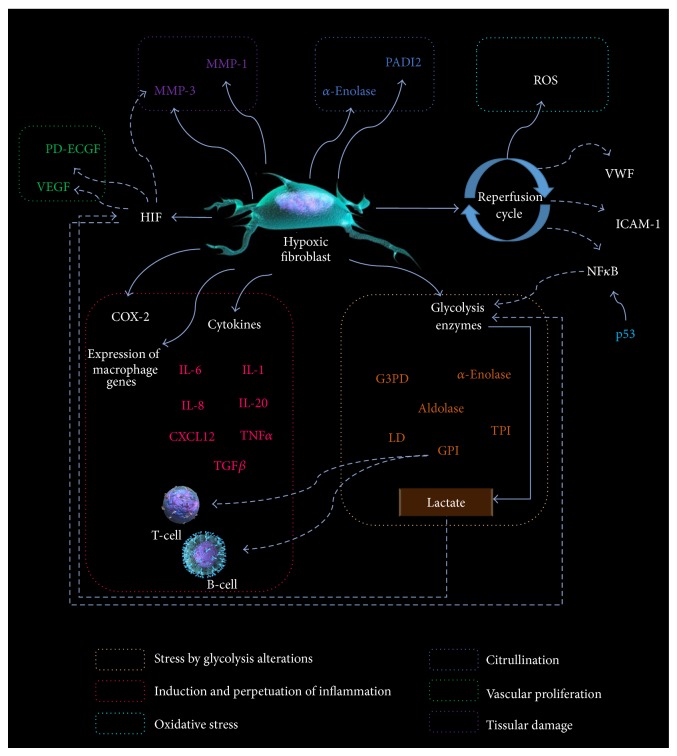
The hypoxic impaired fibroblast in RA. The hypoxia in the rheumatoid synovium induces several phenotypic changes in the fibroblasts; it not only enhances the synthesis of proinflammatory cytokines and metalloproteases, but also induces the glycolysis pathways upregulating the enzymes, which become antigenic because they are abundant in a stressed scenario and eventually are presented by the local APC.

But more importantly, oxygen levels determine several aspects of the metabolism. Energy production by the conversion of glucose to ATP can be obtained either by the aerobic pathway or by glycolysis depending on the oxygen availability. Aerobic oxidation of one glucose molecule generates C0_2_, H_2_0, and 36 to 38 ATP molecules; conversely anaerobic glycolysis generates lactic acid and 2 ATP molecules per glucose molecule. The efficacy of both pathways is out of comparison. Likely anaerobic glycolysis represents a forced second choice, an unpleasant stressing situation; the hypoxic rheumatoid synovium is on it (Figure 3).

As evidence of this metabolic turnaround, critical enzymes of the glycolysis pathway (glyceraldehyde 3-phosphate dehydrogenase and lactate dehydrogenase) are increased in the synovial membrane of patients with RA [131]. HIF is in part responsible for this metabolic phenotype; it upregulates glucose transporters and also induces the synthesis of glycolysis cycle enzymes [132–134]. This preponderance of glycolysis has been confirmed by different methods including resonance magnetic spectroscopy [135]. Additional to HIF, other factors including p53 influence glucose metabolism via ikappaB kinase- (IKK-) nuclear factor and (NF)-kappaB pathways; p53 mutations that suppress its activity have been found in patients with RA [136]; this suppression is thought to enhance glycolytic pathway.

This induction of a glycolytic profile in a setup of stressed cells could result in an effectively costimulated autoimmune presentation of glycolysis related proteins. In that context, hypoxia could drive the immunospecificity of the autoimmune response in RA because it induces several antigenic targets, although those antigens were initially expressed for metabolic correction purposes [137]. For example, Naughton [138] suggested that the anaerobic metabolism induced by the hypoxia increases the expression of glycose-6-phosphate isomerase (GPI). In RA, this enzyme is recognized as an antigen by T- and B-cells [131] and as hypoxia persists, so does the induction of GPI, and that creates a mechanism that perpetuates the hypoxia-induced inflammation. Is that a clue to the almost always relapsing disease activity? GPI performs several roles aside from being a glucose-6-phosphate catalyzer; it is also a maturation factor and a neuroleukin [139]; it is present in synovial fluid from patients with RA [140] in both an isolated metabolically active and also immune-complexed isoform [141]. Patients with RA have antibodies targeting GPI both in serum and synovial fluid and they are clinically meaningful [142, 143], and overexpression of GPI in the synovium has been described as well [144]. However, antibodies to GPI are neither exclusive nor predominant in RA [142]. Therefore hypoxia and secondary glycolysis associated with cellular stress may play a role in the genesis of other arthritides.

*α*-Enolase is another highly conserved catalytic enzyme of the glycolytic cycle and has also been pointed as a potential antigen in RA. Antibodies to *α*-enolase have shown a specificity of 97.1%, in RA patients [145]; furthermore, citrullinated *α*-enolase is even more immunogenic [146]. *α*-Enolase is very phylogenetically preserved and a cross-reactive scenario with the bacterial enolases has been inferred [147]—a lá hsp. In the same direction, other enzymes of the glycolytic pathway, aldolase and the triose phosphate isomerase, have been also defined as autoantigens in patients with RA [137]. Furthermore not only the enzymes but also some glycolysis substrates such as pyruvic acid [148] enhance angiogenesis and lactate carries some proinflammatory effects since it increases VEGF and HIF [149].

A desirable outcome for hypoxia as a real pathogenic process would be whether it could be linked to citrullination under a feasible and realistic mechanism. In that regard, del Rey et al. [150] analyzed the transcriptional response of normal synovial fibroblasts and those obtained from RA patients in normoxic and hypoxic conditions. Of interest, the upregulated transcripts in RA fibroblasts under hypoxia included several enzymes linked to metabolic pathways (mostly for lipids and carbohydrate) and many signaling pathways were preferentially upregulated as well. Although not in the focus of the authors, peptidylarginine deiminase type II (PADI2) and *α*-enolase both linked to the process of citrullinated antigens were upregulated under hypoxic conditions, and also IL-6 as well as several cytokines and proinflammatory mediators. The link between hypoxia and citrullination is not defined in the synovium but such link has been described in astrocytes where PADI2 [151] upregulates citrullination under hypoxic conditions; it is reasonable to assume it could happen in the synovium.

However despite all the negative probed effects that hypoxia induces in the synovium, it would be interesting to ask: why is RA not more common in patients with systemic hypoxia, even chronic progressive hypoxia, such as chronic pulmonary diseases? Probably this is because in most scenarios the level of cellular discomfort induced by the hypoxia is manageable. The synovial fibroblast seems to be unable to deal with a real threat, because the synovium is indeed hypoxic; therefore it is an impaired cell—not a paranoid, but as mentioned by Jeon et al. [107] hypoxia precedes inflammation at least in the collagen-induced rat model of arthritis, so we can assume this sequence of events could be a possibility in the human disease.

Hypoxia has been explained in the rheumatoid synovium to be a consequence of the rapid cellular proliferation induced by the inflammatory response; if the opposite could be true, that is, the cellular proliferation is induced by the inflammatory response caused by the hypoxia, then two fundamental factors are to be defined: first, what explains the localized synovial hypoxia in an otherwise normoxic subject and, second, which mechanisms that make the cells endure the hypoxia are defective in the synovial fibroblasts in patients with RA. Very probably both answers lie far from the immune response we have been focused on as the explanation for the pathogenesis of this disease, as the relationship between tobacco and RA does.

#### 5.1.3. Impaired Keratinocyte in Psoriasis (and Psoriasiform) Lesions

Psoriasis is chronic inflammatory skin disorder caused by keratinocyte hyperproliferation, angiogenesis, and infiltration of the skin by immune cells; an autoimmune background has been inferred. The role of the keratinocyte has been considered as secondary to that of T-cells, which are recalled as the central player; however the keratinocyte is anything but a passive actor in the skin homeostasis, with inflammation included.

Psoriasis is known to have a high genetic predisposal and several candidate genes have been described. Among those, PSORS1 explains 50% of the genetic variance and HLA-Cw6 (specifically Cw^*^0602) seems to be the stronger link; however the possibility that other alleles within the same locus cosegregate with Cw^*^0602 cannot be ruled out, and at this time no clear role for HLA-Cw6 in the pathogenesis of psoriasis has been inferred. Aside from its physiological role as an antigen presenter no aberrant function of HLA-Cw6 has been described and no differences have been found between cases and controls in the sequence nor in regard to epigenetic regulation [152]. Conversely other genes within the PSORS1 locus have gained interest lately. In the same PSORS1, HLA-Cw6 colocalizes with two other genes: corneodesmosin and coiled-coil alpha-helical rod protein 1 (CCHCR1); there is very strong linkage disequilibrium between them, and that limits our possibility to understand their individual isolated effect.

CCHCR1 is expressed in psoriatic skin in counterpart to normal skin; transgenic mice with two variants of CCHCR1 do not express a psoriasis picture but present abnormal keratinocyte proliferation suggesting a potential role for this gene in this process [153]. CCHCR1 regulates the synthesis of steroids from cholesterol, and altered lipid metabolism has been detected in uninvolved skin from psoriatic patients [154].

On the other hand, corneodesmosin SNPs do confer susceptibility to psoriasis in humans [152, 155]. Corneodesmosin is an extracellular protein that integrates into desmosomes before their cornification and is responsible for the corneocyte adhesion and the conformation of the corneodesmosome [156, 157] which is fundamental in the integrity of corneal stratum. Corneodesmosin is expressed in abundance in the psoriatic skin—probably because it is defective and therefore upregulated—in counterpart to normal skin and its expression in psoriasis differs also from other skin diseases associated with proliferation. The complete absence of corneodesmosin leads to the peeling skin disease [158] which differs from psoriasis so the variants of corneodesmosin associated with psoriasis likely remain functional at a certain level. Aside from mutations in corneodesmosin the mechanopropioceptive Wnt signaling family is downregulated in psoriatic skin [159]. Psoriasis lesions appear mostly in skin regions under high mechanical demand (i.e., elbows, knees). Defective corneodesmosin impairs the keratinocyte cornification of the most superficial, final strata and built a solid corneum stratum. This corneum stratum constitutes a fundamental barrier to avoid the penetration of several threats. The lack of this optimal corneal stratum very likely will alter the cell environment (pH, mechanical stress, and microbiological ecosystem). Additionally, the absence of steady anchoring will modify the tensile properties of the skin, increasing the mechanical stress in the underlining epithelial layers and likely triggering compensatory mechanisms. Cell proliferation could be one of them; the induced cellular distress could recruit inflammatory cells throughout danger signals.

The endurance of the keratinocyte is conferred by the action of several constitutive proteins; cytoskeletal components are evidently among them. The cytokeratin 1 knockout mice present inflammatory disease resembling psoriasis or atopic eczema [160]. Cytokeratin 1 (CK1) is specifically expressed in the spinous and granular layers of the epidermis; therefore it is assumed to play a role in the differentiation of the keratinocytes to their final stages of the keratinocytes. In the CK1^−/−^ several cytoskeletal abnormalities in intermediate filaments are observed, as well as a defective inside-out barrier—twice the loss of transdermal water. The absence of CK1 induced the upregulation of 163 genes and downregulation of 2. Amongst the upregulated proteins, some are known to play a role in the inflammatory process: IL-1b, IL-18, IL-33, defensins, and S100 proteins; IL-18 secretion plays a prime role in the inflammatory process. Aside from inflammatory genes some epidermal barrier genes (SPRRs, S100, KLK) are upregulated as well suggesting an attempt to strengthen the weakened skin barrier. Lipid metabolism genes are upregulated as well. The authors conclude that integer CK1 precludes the abnormal liberation of IL-18. In the same journal an editorial to this paper by Hobbs et al. [161] mentions that, based on the results, CK1 plays a role regulating the innate immune response. Although any perspective is valid, it should be noted the real capacity of the keratinocyte to induce and sustain a chronic inflammatory process without detectable abnormalities in the immune response (innate or adaptive). It probably indicates, as well, that our quest to understand chronic inflammatory diseases should also be focused in tissular cell dysfunction and not only in the immune cells and processes.

Following the same line of thought, understanding the potential roles for the keratinocyte in the pathogenesis of psoriasis, we can take a look into the transgenic murine induction of IL-17C in the keratinocytes [25]. In this model, cardinal psoriasiform features are replicated, including the abnormal thickening and proliferation of the epidermis and also the infiltration of CD4+ T-cells. In humans, the psoriasis inflammatory process is considered to be driven by activated T-cells and among them the TH17 subtype prevails. However, specific analysis of IL-17 subtypes produced at the psoriatic plaque and also the cell that produces them [162] has shown that the predominant isoform is IL-17C in 125/1 ratio with IL-17A, and the source is the keratinocyte, not the T-cell. From the murine model we can conclude that the production of IL-17 from the keratinocytes is all it takes to induce psoriasis; the rest of events (even with the involvement of the adaptive immune cells) are a consequence of the downstream effects of IL-17. From the analysis of the human psoriatic plaque we can conclude that the keratinocyte is indeed the source of IL-17. We can ask what is abnormal, the T-cell or the keratinocyte? Or additionally we could state that once you convince a keratinocyte to produce IL-17 little else is needed to induce psoriasis; T-cells are just a part of the choreography.

Another murine model for psoriasis also induces the disease solely altering genes in the keratinocyte. The KO/transgenic mice that selectively express* JunB/c-jun *genes in the keratinocytes also induce lesions that resemble psoriasis [163].* JunB *is antagonic to* c-jun* and the signaling cascade they relate to is not at all specific to the response to immune mediator but it mostly acts as a cellular housekeeping signaling system.* JunB/c-jun* are related to a diversity of cellular functions such as the proliferation or to the reaction in stressful scenarios; therefore we can conclude that this signaling circuit deals with danger scenarios.* JunB/c-jun *mediate responses to several homeostatic systems including calcium channels or hormones; it binds directly to DNA activating the AP-1 transcription factor and is antagonized by* c-jun*. In a very interesting scenario the authors crossed these transgenic* JunB/c-jun *mice with the T- and B-cell deficient RAG2^−/−^ model. As a relevant finding, the cardinal macroscopic and microscopic features of psoriasis persist, although the severity of the infiltrate is milder. The chemokine/cytokine environment in the psoriatic plaques did not differ between both groups of mice whether RAG genes were functional, therefore implying that T-cells are not essential to establish the chemokine/cytokine profile observed in psoriasis. Once again the adaptive immune response is the microphone but not the voice.

Also in another keratinocyte KO model, the deletion of Evi/W1 and subsequent blockade of the WNT pathway also induces a psoriasiform disorder [164]. After the deletion of Evi, several cytokeratins are altered and several proinflammatory cytokines including IL-1, IL-13, and IL-17F, as well as several chemokines, were upregulated. The epidermal barrier was abnormal as evidenced by both protocols: increased dye penetration and transepidermal water loss, suggesting a stressful scenario for the keratinocytes. The onset of the barrier abnormalities coincided with the onset of inflammation suggesting a time link, probably a causal relationship and opening a door for danger signals from stressed keratinocytes to explain the inflammatory phenotype. Infection due to increased barrier permeability was ruled out as an explanation for the inflammatory infiltrates.

So we can conclude that if a significant dysfunction mounts on the skin barrier, inflammation is a likely consequence, and the keratinocyte is able to drive an eventual chronic immune response even in the absence of adaptive immune response cells, and in the case of its presence the keratinocyte is capable to instruct them.

#### 5.1.4. Additional Examples of Impaired Cells

Duchene's dystrophy (DD) is a lethal muscle disease affecting young boys. It is not a rheumatic disorder but can help us to understand the link between an impaired cell with chronic inflammation and also with progressive destruction. DD is explained in every case by the punctual mutation in one protein, dystrophin. No other pathogenic feature aside from a nonfunctional dystrophin is required to explain the disease; no abnormalities in the immune response of the patients with DD have been demonstrated. Dystrophin is an intracellular rod-shaped protein which binds the actin in the sarcomeres and connects it to a multiproteic membrane based complex; the integrity of this complex (dystrophin associated protein complex) is fundamental to avoid mechanical stress damage in the sarcolemma induced by the contractile sarcomeres [165]. Dystrophic muscle fibers are altered in their morphology presenting a progressive reduction in their caliber and an altered structure; they are repopulated by satellite precursor cells, which are depleted as the disease progresses; indeed this depletion is what explains the irreversible muscle wasting.

Although DD was considered merely as a structural disease, recently it was found that the progressive muscular dysfunction in these patients has also an autoimmune inflammatory component. It is understood that aberrant mechanotransduction stimulates inflammatory cascades [166, 167]. Indeed elevated cell infiltration and expression of immunoregulatory molecules are assumed. The infiltrating cells are mostly T-cells (62%) evidencing a prime role for the adaptive immune response, and also macrophages (38%). T-cells are predominantly CD4+ and not CD8+ [168–171]. Infiltrating T-cells respond in a polyclonal fashion to uncharacterized antigens, APC appear in the muscle at a very early age (6–12 months). Clonal analysis of T-cells shows difference in the receptor genes if compared to polymyositis suggesting a particular profile of immune response [172, 173]. Clear mutation of structural proteins and inflammation grow around [174, 175]; indeed a TLR7 pathway of signaling has been proposed [175]; several chemokines are upregulated and correlate with inflammatory infiltrates and they are mostly produced by macrophagic lineage (IL1, TNF-*α*) [176, 177]. Indeed a murine model for muscle dystrophy mdx if bred with TNF deficient mice (mdx/TNF ^−/−^) has significantly lower pathologic markers in the muscles although the disease progresses but at a slower rate. As we can see many of the aspects described in the immunopathogenic process in the DD recall the immune infiltrates in rheumatic autoimmune diseases.

DD is included in this paper despite not being rheumatic to illustrate two key elements in the relationship between the impaired cell and the chronic destructive inflammatory process. First, despite being a genetic prenatal abnormality, the expression of the disease appears later in life despite a period of apparent normality; during this period compensatory mechanisms preclude the abnormality; DD is an early disease but several other dystrophies appear at later stages in life. Second, inflammation is a consequence of a tissular cell dysfunction (the myocyte) with no role in the immune response; however its dysfunction eventually involves even the adaptive immune response in an antigenic unspecific profile. The stressful unresolved scenario that myocytes face in the absence of functional dystrophin eventually triggers the inflammatory process and the immune response and both explain tissular damage.

### 5.2. Model B: The Paranoid Cell

In this model, the cell is not impaired metabolically nor structurally and from a theoretical perspective is normal in regard to its structure, but nevertheless there is a deficit in the calibration between the sensor mechanisms and those exerting the outcome messaging signals; the threshold is out of tune. That is, either the cell is more sensitive than required to a specific stimulus (mechanic, temperature, and hypoxia) or a normally sensed stimulus is transferred into an exaggerated response. In both circumstances even a normal, physiological stimulus (like commensal flora) drives the cell to the perennial annoyance status. The cell is considered to be paranoid because the release of danger signals occurs in the absence of a real threat. Defective anti-inflammatory receptors or mediators could explain this paranoid scenario. Below we will discuss two different scenarios to illustrate the theory of a paranoid cell.

#### 5.2.1. The Overstimulated Intestinal Epithelial Cell in Crohn's Disease

One example of a paranoid cell could be the intestinal epithelial cell in Crohn's disease (CD). CD is a multifactorial inflammatory disorder of the gastrointestinal tract. Uncontrolled mucosal inflammatory response targeting intestinal flora plays a role in the pathogenesis of the disease. Mutations on the nucleotide-binding oligomerization domain 2 (NOD2) gene confer strong genetic risk for CD. However the mechanisms by which this mutation predisposes to intestinal inflammation remain controversial. NOD2 is a key PRR in innate immune responses; it is encoded by the CARD15 gene and its polymorphisms are the single more important risk factor to develop CD [178]. NOD2 contains a NOD domain linked on its C-terminal side to a leucine-rich repeat domain (LRR) which is responsible for interacting with the microbial ligands (mainly peptidoglycan), and in its N-terminal side it has 2 caspase recruitment domains (CARDs) which are responsible for the downstream signaling interactions [179, 180]. NOD2 is expressed on dendritic cells and also in epithelial cells in the gut including Paneth's cells at the bottom of the crypts. Interestingly, NOD2 stimulation with microbial ligands exerts weaker responses than those of observed with TLR stimulation [181] and therefore NOD2 is considered to be a downregulator of several TLR related responses. NOD2 has been proposed as a relevant protective molecule against the invasiveness of certain bacteria including* L. monocytogenes* or* H. hepaticus*; NOD2 deficient mice lack an adequate production of cryptins including defensins [182] which are a critical regulatory mechanism in the epithelial microbial interface; however a reduction in defensins has been reported in CD patients without NOD2 mutations [183]. Mutations of the NOD2 protein in CD generate a hypofunctional protein explained by lower proinflammatory responses after the binding of NOD2 to the microbial ligands; most CD related polymorphisms are located in the LRR region of NOD2 therefore reducing its responsiveness to them. It has been traditionally assumed that NOD2 deficient response impairs the bactericidal response of the gut mucosa and explains exaggerated inflammation to compensate its lack of efficiency; in that perspective the epithelial gut cell would be an impaired cell; however recent evidence at some point contradicts that premise unveiling a potential role as a downregulator of the local mucosal inflammatory response.

**Figure 4:**
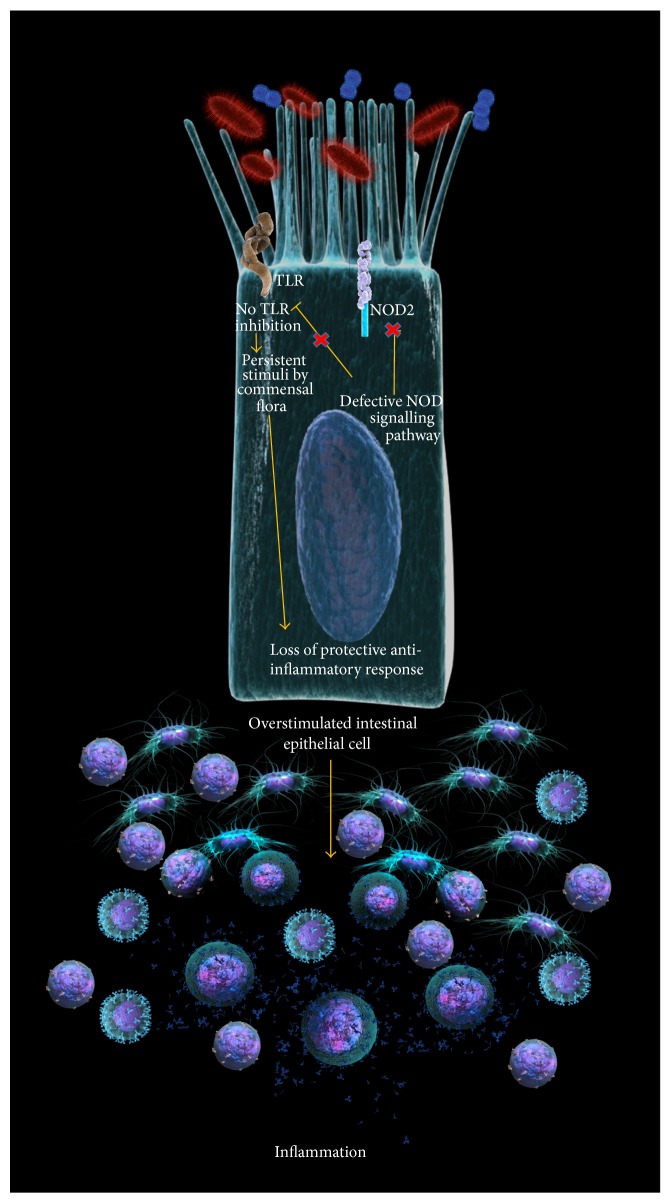
The paranoid overstimulated intestinal epithelial cell in Crohn's disease. The deficiency in NOD2 inhibits an anti-inflammatory mechanism that impedes TLR2 from continual signaling if in contact with its bacterial wall ligands. The loss of this compensatory anti-inflammatory mechanism generates uncontrolled inflammation based on a threat that is not real, because the commensal flora does not harm.

In that regard the risk for infection is not that real. The role of NOD2 as a regulatory molecule is crucial in the GI tract since the perennial and abundant presence of bacterial components which are ligands to NOD, TLR, and other PRR has the potential to trigger a continuous stimulation of the immune elements of the intestinal mucosa. It is possible to assume that defective NOD2 function could explain an impaired regulation of TLR responses specially TLR2 [181]. It is also hypothesized that the inferred immunodeficiency conferred by a defective NOD2 is arguable and that the presence of bacteria or bacterial components in the lamina propria has not been proved to trigger the inflammation in CD. In the other hand, the continuous stimulation of NOD2 with muramyl peptide could tolerize macrophages previously stimulated with either TLR2 or 4 [184, 185]; therefore, defective tolerizing proteins such as NOD2 could induce a perpetual inflammatory status despite; in this case, the threat of infection (i.e., bacterial invasion) is not real (Figure 4).

At this point the specific mechanisms that explains how NOD2 confers tolerance to bacterial cell wall components providing a protective scenario against the unleashed activation of TLR [186] are unclear. The ligation of NOD2 to the muramyl peptide dipeptide induces rapid degradation of NOD2 via ubiquitination and proteasomal degradation but some mediators could influence this degradation.

Persistent unregulated TLR2 stimulation may result in perennial inflammation despite the absence of a specific infection. The immune cells are stimulated with no specific purpose. Therefore since the threat is not real, the cell is paranoid; inflammations arise as the consequence of a defective immunomodulator mechanism, an anti-inflammatory mechanism crucial for the everyday coexistence with nonpathogenic intestinal flora.

#### 5.2.2. The Paranoid Osteotenocyte in Ankylosing Spondylitis

Ankylosing spondylitis (AS) is an inflammatory disease characterized by the ossification of entheses of the spine at latter stages of the disease; it is highly associated to HLA-B27. Enthesitis is the hallmark of AS and of the group where it belongs, the spondyloarthritides (SpA). Benjamin and McGonagle provided an outstanding review of the structural and functional aspects of the enthesis affected in SpA [187]. In the authors' opinion, two relevant factors explain the selective pattern of SpA enthesitis. The first factor is the presence of fibrocartilage (FC) in the enthesis in the interface between the tendon and the bone attachment; all of the larger entheses have it. The second factor is a high mechanical demand explaining a mechanical stress (MS).

The pattern of ossification in the spine of AS patients also suggests a role for mechanical stimuli. The lower segments of the lumbar spine are the first to ossify and once they become rigid, the tensile demands for the ligaments in the upper segments increase. This relation between the ossification and mechanical stimuli and its relative dissociation to the inflammation have also been described [188, 189]; indeed the roles of signaling systems such as WNT [190–192] and bone morphogenetic proteins [193, 194] have being selected as responsible mediators to drive the osteogenic stimuli.

A role for MS as a pathogenic factor has been assessed in animal models for SpA. In the paper from Jacques et al. [195] the authors selected a murine model with increased and deregulated expression of TNF-*α* (TNF^ΔARE^) which develops arthritis and ileitis. The authors prove the relevance of the MS over the entheses to explain the onset of arthritis, enthesitis, and sacroiliac fusion, with all of those being basic features of AS. To do so, the authors demonstrated higher inflammation and proliferation scores in the group of mice which were kept in normal gait against a comparative group that were suspended from the tail before the arthritis began. Interestingly in this model (as with the psoriasis IL-17c being transgenic) the absence of mature T- and B-cells made no difference in the severity of the enthesitis; mostly stromal cells (fibroblasts, chondrocytes) explained the complete pathogenic picture. The authors conclude that MS triggers the inflammation; it begins in the entheseal insertion and suspending the mice from the tail precludes the inflammation in the rear limbs.

The pathogenesis of the SpA involves MS very likely. But is the SpA typical on high demand athletes or obese persons? Or are they more prevalent in persons over a high weight loading demand? None of the previous is correct. Under normal circumstances, the enthesis responds to excessive weight load reinforcing its structure. When an enthesis is overdemanded morphological changes become evident; noninflammatory ossification of the muscle insertions is seen in athletes.

Entheses are designed to feel and resist the mechanical stress; they are designed to adapt and respond to mechanical stress, so we could better hypothesize that the link between inflammation and MS is related to an abnormal perception of MS; lesions in the tendinous structures (such as ruptures) are not described in SpA patients so we have no evidence that the SpA patients' tendons are weaker than those in normal persons and therefore under higher MS. Microscopic microfractures in the entheseal bone have been proposed as an indicator of impairment [196], but the histological samples from SpA do not show as a rule evidence of weakened tendons or enthesis, weakened entheses have been reported neither in the transgenic HLA-B27 nor in other SpA rodent models.

Likely, the problem is not the MS itself, but an abnormal perception of it; but in this case, the cell (the osteotenocyte) is not impaired, but paranoid. An additional element on this paranoid behavior of the osteotenocyte comes from the fact that the bone formation exceeds by far the actual requirements of the remodeling process. It does so, up to a point where the ossification itself becomes a problem. The mechanical demand is not real; it is the cell's perception of it which is pathogenic.

Genetic risk conferring genes for AS or SpA in general do have very little to do with structural proteins and HLA-B27 does not play a known role in the buildup of the enthesis or bone.

In order to understand the role of abnormal mechanosensing to the pathogenesis of SpA it has to be connected somehow to HLA-B27. HLA-B27 has been described as a cardinal association to AS [197, 198]. Understanding the precise role of HLA-B27 in the pathogenesis of SpA, however, is far from being clear. The HLA-B27/*β*2 microglobulin transgenic rat model rat model replicates some key features of SpA [199]; therefore it is assumed that HLA-B27 is not an epiphenomenon.

The initial approach to understand the pathogenic role of HLA-B27 was based on its role as an antigen-presenting molecule to CD8 T-cells. This hypothesis is further supported by the fact that polymorphisms in antigen processing related proteins such as ERAP1 [200] confer also an increased risk to develop AS. However CD8+ T-cells do not predominate at biopsy sites in patients with AS [201, 202], and the CD8 knockout transgenic HLAB27/ß2 microglobulin rat presents the SpA-like disease with no change in its severity [203], so the role of HLA-B27 as antigen presenter to CD8 is questioned.

It was eventually known that HLA-B27 can take several molecular conformations [204–208] and this expands its potential interactions with other cellular populations beyond CD8. Free heavy chains interact with a diversity of ligands like killer cells immunoglobulin-like receptors (KIR), which are expressed by CD4, CD8, and natural killer cells. Two recent papers [209, 210] conclude that HLA-B27^*^05 induces more KIR3DL2 reactive polymers than HLA-B27^*^09 and also than HLA-A3. These HLA/KIR interactions are limited neither to SpA [211, 212] nor to HLA-B27 [213–215]. However, does MS influence the presence or proportion of these pathogenic variants?

Aside from the intercellular interactions of HLA-B27 canonical or not, its intracellular posttranscriptional processing has been pointed as a potential source for cellular annoyance. The folding of HLA-B27 takes longer time than other HLA-class I subtypes [216]; its persistence in the endoplasmic reticulum is prolonged; HLA-B27 binds to several chaperones including BiP and induces a stress response. Although this slow intracellular traffic is known to be specific neither to HLA-B27 nor to those subtypes conferring AS susceptibility [217]. However, does MS further delay the folding and posttranscriptional handling of HLA-B27? Or does MS share signaling mediators with those of the delayed folding response opening a door for potential synergy? Would not it be great to analyze the intracellular trafficking of HLA-B27 under the scope of in vitro models of mechanical sheer stress?

But in the end of the day, neither the HLA-B27 multifaceted surface expression, nor the possibilities to interact with a diversity of ligands, nor its annoying, prolonged intracellular presence is exclusive for the HLA-B27 AS-risk-conferring alleles. So probably its uniqueness as a AS susceptibility conferring gene might be in its specific behavior in the entheseal cell under MS and the type of cellular discomfort that is produced in the diseased patient in every step and every bending. As can be inferred by the Jacques et al. [195] model probably T- or B-cells are not required.

Of interest, maybe other cardinal features in AS patients such as acute anterior uveitis and aortitis might be partially explained by the high mechanical demand on both organs instead of its antigenic composition. Very likely, both cell strains share the mechanical stress/HLA-B27 paranoid combination with the osteotenocyte.

## 6. Final Remark

*IL-17, Is It the Emblematic Mediator of the Ruling T-Cells and the Adaptive Immune Response? Or Is It Every Tissular Cell's Danger T-Cell Herding? Every Cell Seems to Be Able to Produce It, but Who Is in Command?* TH17 cells seem to be in the center of our current understanding for the pathogenesis of several rheumatic diseases, making us forget about TH1/TH2 perennial dichotomy; basically every updated analysis of the pathogenic pictures of rheumatic diseases deals with TH17 cells.

Th17 preponderance has been inferred by the presence of IL-17 as a relevant proinflammatory mediator, and IL-17 has gained a growing interest, both as a key pathogenic element and also as a potential therapeutic target in rheumatic diseases including rheumatoid arthritis [218–220] and spondyloarthritis [221] including among them psoriatic arthritis [222, 223].

But IL-17, though traditionally attributed to T-cells (more precisely TH17), is far from being restricted to them. Aside from macrophages [224, 225], neutrophils [226], and Paneth cells [227] several cell lineages including epitheliums produce it. The catalog include keratinocytes [25], gingival cells [228], lung alveolar [229], respiratory airway [230, 231], and nasal [232] epithelia, endometrium (both epithelial and stromal cells) [233], seminal vesicles glands epithelia [234], colonic epithelial cells [235], and mammary gland acini [236] cells.

IL-17 is not a single molecule but a family and it includes 6 subtypes; IL-17 (also called IL-17A) and IL-17F are those attributed to be produced by TH17; however several of the above referenced tissues produce specifically IL-17A and not only the “epithelial” IL-17C.

Indeed, going back to rheumatic diseases in the synovial membrane where TH17 response is considered pivotal, the neutrophils [237] and the mast cells are indeed the principal source of IL-17 for the case of rheumatoid arthritis [238, 239] and mast cells are also relevant IL-17 secretors in the spondyloarthritis [240], and keratinocyte derived IL-17C is the predominant isoform in psoriatic skin lesions [171].

So, who drives the IL-17 train?

## 7. Conclusion

Although significant advances in our understanding of the pathogenesis of rheumatic diseases are evident, our knowledge of their precise etiology remains evasive. Most of our current strategies have focused on the study of the immune response cells and processes related to them; however, an increasingly important role of the tissular cells and the disturbance in their basic functions is being detected as we dig in that field in our quest to understand rheumatic diseases.

From the danger model perspective we can state that whatever surrounds or lies within a harmed tissue is potentially antigenic; clonal deletion from the thymus biases the response toward foreign structures, however in a far from perfect fashion.

Autoimmunity is a tree of immune dysregulation planted in a soil of defective cell housekeeping.
